# Pain, inconvenience and blame: defining work-related injuries in the veterinary workplace

**DOI:** 10.1093/occmed/kqae068

**Published:** 2024-08-12

**Authors:** T Furtado, M Whiting, I Schofield, R Jackson, J S P Tulloch

**Affiliations:** Institute of Infection, Veterinary and Ecological Sciences, University of Liverpool, Liverpool, CH64 7TE, UK; CVS UK Ltd, Diss, IP22 4ER, UK; CVS UK Ltd, Diss, IP22 4ER, UK; CVS UK Ltd, Diss, IP22 4ER, UK; Institute of Infection, Veterinary and Ecological Sciences, University of Liverpool, Liverpool, CH64 7TE, UK

## Abstract

**Background:**

The veterinary workplace carries a high risk of staff accidents and injuries, yet there is scant research exploring it in comparison with other comparable fields, such as human medicine.

**Aims:**

To understand how veterinary professionals define injuries and to understand what injuries they do, or do not, deem reportable.

**Methods:**

A cross-sectional survey comprising demographic questions and open-text questions was shared with veterinary practice staff across the UK. Data were analysed descriptively and using an inductive content analysis.

**Results:**

There were 740 respondents, who were broadly representative of the veterinary profession. There were differences in how injuries were defined; for example, small animal veterinarians expected injuries to involve blood, while equine and production animal veterinarians were more likely to expect injuries to reduce their ability to perform work and require medical treatment. Many suggested that ‘all’ workplace injuries should be reported; however, ‘minor’ injuries were often overlooked, for example, needlestick injuries did not always meet the criteria of being an ‘injury’. Injuries caused by staff themselves (e.g. trips) were less likely to be reported than injuries that could be blamed on an external factor (e.g. dog bite).

**Conclusions:**

Collectively, the data suggest a wide-ranging perception of risk of injury in practice, with some harms seen as ‘everyday norms’. Veterinary practices should interpret their injury statistics with a high degree of caution. They should explore the microcultures within their practices relating to worker perception of risk, injury and barriers to reporting.

## Teaser

This survey highlights that the veterinary profession defines work-related injuries in a highly varied and nuanced way. Equine and production animal veterinarians define an event as injury if it reduces their ability to perform work or results in medical treatment. Injuries have become normalized and accepted as an everyday occurrence; resulting in many injuries not being reported. This work shows that the veterinary profession has a concerning attitude to work-related injuries.

**Key learning points**

**What is already known about this subject:**

- The veterinary industry has one of the highest case rates of non-fatal occupation injuries and illnesses per full-time worker.
- In the USA, no other industry is higher; it is almost five times higher than the national average.
- Little research has explored how injuries are perceived or their context.

**What this study adds**

- This study shows clear divisions within different veterinary sectors and job roles, in the perception of injuries, and how injuries are perceived.
- Equine and production animal veterinarians have a high threshold before acknowledging that an incident is a work-related injury.
- Many consider hazards, such as horse kicks, to be everyday risks and so do not deem them worthy of reporting.

**What impact this may have on practice or policy**

- To contextualize any epidemiological research into veterinary workplace injuries, one must understand how injuries are perceived.
- The discordance in definition needs to be accounted for when interpreting company or national injury reporting figures.
- Such figures are only likely to represent the most severe of injuries and may not reflect the overall injury burden of the veterinary workplace.

## Introduction

The veterinary industry has one of the highest rates of work-related injuries. In the USA, it has the highest levels of non-fatal occupational injuries and illness (13.8 cases per 100 full-time workers in 2021), almost five times higher than the national average [1,2]. In the UK, being an equine veterinarian has been deemed the most dangerous civilian profession [3]. Potential hazards include the physical working environment, machinery, sharp equipment, toxic materials, medications and animals [4,5]. However, veterinary workplace injuries have received little sociological and epidemiological research attention, in comparison with other medical settings.

Exploration of work-related injuries must start by considering how industry-specific employees define a work-related injury. The Center for Disease Control and Prevention (CDC) defines an injury as ‘A bodily harm resulting from severe exposure to an external force or substance (mechanical, thermal, electrical, chemical, or radiant) or a submersion. This bodily harm can be unintentional or violence-related’ [6]. The Health and Safety Executive states that ‘An accident is “work-related” if any of the following played a significant role: the way the work was carried out; any machinery, plant, substances or equipment used for the work or, the condition of the site or premises where the accident happened’ [7]. Accident-reporting systems rely on individuals being able to identify when a work-related accident has occurred and then report it. If an employee utilizes a different work-related injury definition, then reporting rates will not reflect the true extent of injuries.

Differing job roles in an industry may perceive different levels of acceptable risk [8], and how they define an injury [9]. Additionally, emotional intelligence is associated with lower levels of injury. Suggesting that psychological and emotional traits might impact perceptions of safe practices and behaviour [10]. Different workplace activities, professional values and work cultures will exist within any given industry and workplace, contributing to what people perceive to be an injury and thus how people recognize and respond to work-related risk.

The reasons for injury under-reporting include a lack of trust that change will occur; lack of time; lack of familiarity with reporting practices; concern over getting colleagues in trouble and concern regarding blame [11,12]. Veterinary work is known to be both highly risky and highly specific; therefore, we might expect veterinary perception of accident reporting to differ from other industries. However, beyond establishing that veterinary work presents a high level of risk, particularly for production animal and equine veterinarians [2–4], the veterinary industry has been predominantly overlooked.

This study aims to understand how the veterinary professions define injuries and what type of injury they do or do not, deem reportable. This information can be used to support veterinary practices in exploring their approaches to risk and accident identification and reporting, and thus create safer workspaces.

## Methods

An online questionnaire was developed, comprised of categorical, numerical and open-ended questions. Participants were asked about personal demographics and their work (i.e. the type of practice and job role). They were asked to provide their definition of a work-related injury, to describe a work-related injury that they felt needed reporting and one that did not. The questionnaire was piloted with a sample of veterinary professionals.

The survey was distributed to all UK employees of CVS UK Ltd (CVS), which comprises approximately 500 veterinary practices, through an internal newsletter and was open for 3 months between 6 December 2022 and 6 March 2023. To enhance recruitment, an incentive of a year’s supply of snacks for five randomly selected practices was provided.

All responses were stratified by job role. Descriptive statistics were performed to describe the respondent population, and open-ended questions were analysed using inductive qualitative content analysis [13,14], which provides a flexible approach combining elements of qualitative analysis with quantitative components. Each response was initially read, whilst notes describing content categories were created, then each response was considered in isolation and was codified to reflect the information within that item. As content codes were created, they were combined, revised and deleted. This iterative process created a final set of codes which ensured internal validity of content in the codes across the dataset. Instances of each code occurring were then counted to facilitate a quantitative content comparison.

Data masking was used to protect the personally identifiable information of respondents. The study received ethical approval from the University of Liverpool Veterinary Research Ethics Committee (VREC1256).

## Results

Over 1000 individuals (*n* = 1102) consented to the survey, with 740 (67%) complete responses available for analysis. Veterinarians were the most prevalent job role (34%), of whom 75% were companion animal veterinarians. Other roles included veterinary nurses (28%), administrators (16%), receptionists (14%) and companion animal Patient Care Assistants (PCA) (8%) (Table 1).

**Table 1.** Veterinary work-related injury survey respondent demographics

	Equine veterinarian *n* (%)	Production animal veterinarian *n* (%)	Companion animal veterinarian *n* (%)	Total veterinarians *n* (%)	Companion animal veterinary nurse *n* (%)	Companion animal patient care assistant *n* (%)	Administrator *n* (%)	Receptionist *n* (%)	Total *n* (%)
Sex (% female)	28 (60)	13 (81)	156 (83)	197 (79)	196 (94)	53 (87)	105 (91)	105 (99)	656 (89)
Age									
<20	0 (0)	0 (0)	0 (0)	0 (0)	0 (0)	1 (2)	0 (0)	2 (2)	3 (0)
20–29	7 (15)	6 (38)	43 (23)	56 (22)	60 (29)	33 (54)	11 (10)	35 (33)	195 (26)
30–39	17 (36)	6 (38)	69 (37)	92 (37)	73 (35)	16 (26)	23 (20)	15 (14)	219 (30)
40–49	13 (28)	3 (19)	48 (26)	64 (26)	60 (29)	4 (7)	48 (42)	13 (12)	189 (26)
50–59	7 (15)	1 (6)	18 (10)	26 (10)	11 (5)	5 (8)	25 (22)	23 (22)	90 (12)
60+	3 (6)	0 (0)	9 (5)	12 (5)	4 (2)	2 (3)	8 (7)	18 (17)	44 (6)
Ethnicity									
White	45 (98)	16 (100)	177 (95)	238 (96)	201 (97)	60 (98)	111 (98)	104 (98)	714 (97)
All other ethnic groups combined	1 (2)	0 (0)	9 (5)	10 (4)	6 (3)	1 (2)	2 (2)	2 (2)	21 (3)
Ever had an injury	46 (98)	15 (94)	175 (94)	236 (94)	224 (93)	46 (75)	53 (50)	66 (62)	599 (81)

All respondents who provided their sex also identified with the same gender. Veterinarian respondents were representative of the UK profession in terms of age (median national age 30–39) and ethnicity (4% ethnic minority groups nationally) [15]. However, female respondents were over-represented, 79% females in the survey versus 59% female veterinarians nationally. Companion animal veterinary nurses were broadly representative in terms of sex (97% female nationally), age (median age of 30–39 nationally) and ethnicity (2% ethnic minority groups nationally) [16]. National demographics of companion animal PCA, administrators and receptionists are currently unavailable.

The prevalence of work-related injuries experienced during their career was the highest for veterinarians (94%) and the lowest for administrators (50%). Of the veterinarians, the highest injury prevalence was seen in equine veterinarians (98%).

When asked to define an injury, most participants referred to elements of physical trauma (Table 2). Descriptions coded as ‘physical trauma’ included references to physical injury, damage to the body, or harm: ‘Any incident involving harm to a person’—companion animal veterinary nurse. A minimum of 20% of participants provided greater depth to what they would consider a physical trauma. These codes were divided into three themes, *harm to the individual* (including blood, pain or mental harm), *response required due to harm* (including need for medication/treatment, time for recovery, inability to perform job role) and *accident*. Regarding *harm to the individual*. Many participants explicitly stated they would consider something an injury if there was a high degree of severity: ‘An incident that results in a cut/bruise/wound/fracture/dislocation/pain or damage to my body’—companion animal veterinarian. Pain was seen by some as a core component of how they perceived an injury. ‘Pain’ was used more by equine (35%) and production animal (25%) veterinarians than other roles and tended to be used as a descriptive additive clause to physical harm. ‘An injury is usually an event that happens where your body is hurt and elicits excessive pain/inconvenience’—production animal veterinarian. Mental trauma was referenced by 9% of participants, though always in addition to physical harm: ‘An event which causes tissue or psychological damage’—companion animal veterinarian. Production animal (13%) and companion animal (12%) veterinarians tended to use ‘mental trauma’ more than other roles. None of the participants gave details about the extent or duration of mental trauma that they felt constituted an ‘injury’, which contrasted with the physical trauma definitions.

**Table 2.** Content analysis of the veterinary profession’s definition of an injury (percentages do not add to 100% as respondents’ definitions could include multiple codes)

Injury definition code	Equine veterinarian (*n* = 47) *n* (%)	Production animal veterinarian (*n* = 16) *n* (%)	Companion animal veterinarian (*n* = 187) *n* (%)	Total veterinarians (*n* = 250) *n* (%)	Companion animal veterinary nurse (*n* = 208) *n* (%)	Companion animal patient care assistant (*n* = 61) *n* (%)	Administrator (*n* = 115) *n* (%)	Receptionist (*n* = 106) *n* (%)	Total (*n* = 740) *n* (%)
Harm to the individual									
Physical Trauma	32 (68)	9 (56)	232 (81)	190 (76)	175 (84)	51 (84)	97 (84)	89 (84)	603 (82)
Pain	17 (36)	4 (25)	41 (22)	62 (25)	38 (18)	11 (18)	20 (17)	20 (19)	151 (20)
Mental trauma	4 (9)	2 (13)	23 (12)	29 (12)	15 (7)	3 (5)	9 (8)	10 (9)	66 (9)
Blood	0 (0)	1 (6)	11 (6)	12 (5)	5 (2)	5 (8)	2 (2)	3 (3)	27 (4)
Response required due to harm									
Inability to do work	10 (21)	6 (38)	10 (5)	26 (10)	15 (7)	4 (7)	2 (2)	3 (3)	50 (7)
Need for medical treatment	0 (0)	2 (13)	11 (6)	13 (5)	2 (1)	1 (2)	6 (5)	0 (0)	22 (3)
Time for recovery	3 (6)	0 (0)	4 (2)	7 (3)	4 (2)	1 (2)	1 (1)	1 (1)	14 (2)
Accident	3 (6)	0 (0)	22 (12)	25 (10)	22 (11)	7 (12)	10 (9)	12 (11)	76 (10)

Regarding the theme *responses required due to harm*; many participants described that a core component of their definition of ‘injury’ was the need to take action, for example, to seek medical treatment, take time to recover, or alter their behaviour: ‘Something that requires me to open the first aid box or “take a minute” to recover from while doing my job’—companion animal veterinarian; ‘Where you have hurt yourself and need to go to hospital or it leaves a mark or pain longer than 1 day’—production animal veterinarian. This ‘need for medical treatment’ was most prevalent for production animal veterinarians (13%) compared with equine (0%) and companion animal (6%) veterinarians. The ‘inability to work’ code had the largest difference in prevalence between job roles. It was higher for equine (21%) and production animal (38%) veterinarians compared to less than 10% in all other veterinary roles. It was primarily used in isolation; ‘Any injury that prevents me carrying out my role as a practising veterinary surgeon’—production animal veterinarian. Contrastingly, other job roles tended to combine it with themes, such as pain: ‘Hurt/pain with tissue damage and reduced capacity to work to full potential’—companion animal veterinarian.

Overall, the theme of ‘accident’ was the third most prevalent. This was rarely used in isolation but as a conjunctive to ‘physical trauma’: ‘An injury is something that has caused harm to your body through an accident’—companion animal administrator. This suggests that these participants considered accidental harm differently from harms caused by, for example, negligence.

When asked to describe a work-related injury that they thought needed reporting to their employer, 40% of individuals stated that all work-related injuries should be recorded (Table 3): ‘Any injury sustained in the workplace’—companion animal veterinarian. At least 10% provided details which modified their answer, for example, stating that while all injuries *should* be reported, they would only report an injury if: it was severe; it impacted their ability to work or it required medical treatment. The use of these modifiers was more prevalent in veterinarians than other roles in particular equine and production animal veterinarians. For example: ‘I think you’re supposed to report all of them, but I tend to report only ones that impact my ability to keep working or need intervention to keep working’—companion animal veterinarian. ‘Technically any injury should be reported especially those related to poor maintenance or set-up. I think any injury that may result in seeing a Dr or any time off should be reported’—equine veterinarian. These responses highlight the gap between ways people think they should behave, and how they actually behave, which involved individual estimation of the severity of the injury or requirement of additional support. One participant described her reasons for not reporting in more detail: ‘I’ve perhaps reported some of them but the pace of work is so fast and as I feel it’s not going to change anything for me then I report very few of them’—companion animal veterinarian.

**Table 3.** Content analysis of the veterinary profession’s definition of a work-related injury that they would report to an employer (percentages do not add to 100% as respondents’ definitions could include multiple codes)

Reportable injury code	Equine veterinarian (n = 47) *n* (%)	Production animal veterinarian (*n* = 16) *n* (%)	Companion animal veterinarian (*n* = 187) *n* (%)	Total veterinarians (*n* = 250) *n* (%)	Companion animal veterinary nurse (*n* = 208) *n* (%)	Companion animal patient care assistant (*n* = 61) n (%)	Administrator (*n* = 115) *n* (%)	Receptionist (*n* = 106) *n* (%)	Total (*n* = 740) *n* (%)
All injuries	19 (40)	7 (44)	63 (34)	89 (36)	80 (39)	21 (34)	54 (47)	48 (45)	292 (40)
No injuries	0 (0)	0 (0)	1 (1)	1 (0)	0 (0)	1 (2)	1 (1)	2 (2)	5 (1)
Modifier									
Severity	13 (28)	5 (31)	28 (15)	46 (18)	11 (5)	1 (2)	5 (4)	9 (9)	72 (10)
Impacted ability to work	5 (11)	4 (25)	13 (7)	22 (9)	6 (3)	4 (7)	6 (5)	2 (2)	40 (5)
Medical treatment	3 (6)	1 (6)	6 (3)	10 (4)	6 (3)	1 (2)	6 (5)	3 (3)	26 (4)
Examples									
Bite or scratch	0 (0)	0 (0)	86 (46)	86 (34)	88 (42)	21 (34)	23 (20)	15 (14)	233 (32)
Kick or trample	17 (36)	2 (13)	0 (0)	19 (8)	0 (0)	0 (0)	6 (5)	1 (1)	26 (4)
Slip or fall	0 (0)	0 (0)	10 (5)	10 (4)	15 (7)	5 (8)	11 (10)	23 (22)	64 (9)
Needlestick	0 (0)	1 (6)	17 (9)	18 (7)	13 (6)	4 (7)	4 (4)	4 (4)	43 (6)
Equipment	2 (4)	1 (6)	3 (2)	6 (2)	8 (4)	1 (2)	11 (10)	2 (2)	29 (4)
Musculoskeletal injuries	1 (2)	1 (6)	4 (2)	6 (2)	4 (2)	1 (2)	0 (0)	6 (6)	18 (2)
Burn	0 (0)	0 (0)	0 (0)	0 (0)	2 (1)	3 (5)	0 (0)	2 (2)	7 (1)

When respondents gave examples of injuries that needed reporting, the majority described injuries caused by animals (i.e. bites, kicks) or caused by hazards (i.e. slips, trips and falls). These injuries could be positioned as being caused by other parties, such as the environment, humans or animals: ‘Common injuries in our profession are often bite-related injuries from handling animals’—companion animal veterinary nurse.

When asked to describe a work-related injury they thought did not need reporting, a large proportion of respondents said that they would report all injuries. This was lowest among equine and production animal veterinarians who were more likely to provide specific examples of cases that would not need reporting (Table 4). Veterinarians (26%) would not report if they felt the injury was minor or if they required no medical treatment; equine veterinarians (43%) were the least likely to report injuries that they considered minor: ‘Something that does not require First Aid is not an injury that needs to reporting’—companion animal care assistant. Notably, 8% of veterinarians gave a needlestick injury as an example of an injury that did not need reporting: ‘everyday needle stick injury, occurs quite commonly, as long as not a dangerous drug and minor injury’ (companion animal veterinarian). Companion animal (5%) veterinarians were more likely than other veterinarians to mention a lack of blood as a reason for not reporting. This likely relates to common injury types experienced, as 15% of companion animal veterinarians used bites or scratches as examples of minor injuries, compared with 15% of equine vets who used a kick as an example of an injury that did not need reporting: ‘Vet knocked into wall by horse causing pain initially but no visible bruises or residual pain’—equine veterinarian; ‘In this job role we probably get scratched quite a lot by animals. Unless it’s a really bad/deep scratch I wouldn’t report it to my employer’—companion animal veterinary nurse.

**Table 4.** Content analysis of the veterinary profession’s definition of a work-related injury that they would not report to an employer (percentages do not add to 100% as respondents’ definitions could include multiple codes)

	Equine veterinarian (n = 47) *n* (%)	Production animal veterinarian (*n* = 16) *n* (%)	Companion animal veterinarian (*n* = 187) *n* (%)	Total veterinarians (*n* = 250) *n* (%)	Companion animal veterinary nurse (*n* = 208) *n* (%)	Companion animal patient care assistant (*n* = 61) *n* (%)	Administrator (*n* = 115) *n* (%)	Receptionist (*n* = 106) *n* (%)	Total (*n* = 740) *n* (%)
No injuries	9 (19)	3 (19)	50 (27)	62 (25)	79 (38)	21 (34)	73 (64)	45 (42)	280 (38)
Modifier									
Minor	20 (43)	5 (31)	41 (22)	66 (26)	31 (15)	12 (20)	8 (7)	17 (16)	134 (18)
Self-attribution	4 (9)	3 (19)	15 (8)	22 (9)	25 (12)	2 (3)	5 (4)	7 (7)	61 (8)
No medical treatment	6 (13)	0 (0)	8 (4)	14 (6)	7 (3)	2 (3)	1 (1)	1 (1)	25 (3)
No blood	0 (0)	0 (0)	10 (5)	10 (4)	1 (1)	1 (2)	0 (0)	1 (1)	13 (2)
Example									
Papercut	4 (9)	1 (6)	21 (11)	26 (10)	32 (15)	11 (18)	17 (15)	29 (27)	115 (16)
Bite and scratch	0 (0)	0 (0)	28 (15)	28 (11)	15 (7)	9 (15)	0 (0)	3 (3)	56 (8)
Needlestick	2 (4)	4 (25)	14 (8)	20 (8)	11 (5)	0 (0)	2 (2)	1 (1)	34 (5)
Fall or slip	0 (0)	0 (0)	1 (1)	1 (0)	3 (1)	2 (3)	2 (2)	2 (2)	10 (1)
Kick	7 (15)	1 (6)	0 (0)	8 (3)	1 (1)	0 (0)	0 (0)	0 (0)	9 (1)

Participants frequently described self-assessing the severity of injury and the requirement for behaviour change to avoid future instances, when deciding whether to report an injury. This self-assessment process could include an estimation of whether the accident could have been avoidable, for example, by following protocols or having a clear idea of health and safety procedures: ‘Any injury that you feel is caused by your own lack of judgement/ignorance of health and safety rules and that does not require any treatment or time off work’—companion animal veterinarian. This response also displays an additional theme: self-attribution of injury caused. In contrast to when reported incidents were often the result of interaction with an animal or working environment which caused harm, it was notable that participants considered injuries which did not need reporting to be those which they felt were ‘self-inflicted’. For example, a papercut was the most frequently cited injury perceived as not needing reporting across all groups; other examples included ‘Hurting yourself by being stupid i.e. walking into a door’—companion animal veterinary nurse. There was a clear disparity in the data between incidents caused by another party, which participants felt needed reporting, and injuries perceived as self-attributable which were considered less worthy of reporting.

## Discussion

Work within the veterinary industry is perceived as being frequently harmful with social norms around injury expectations differing across veterinary sectors. These data clarify why there may be differences in injury reporting across the veterinary industry, as people are unlikely to report what they consider to be ‘minor’ or ‘expected’ injuries. Nevertheless, there was considerable variation within each group, suggesting that some professional cultures may not perceive each of these incidents to be ‘expected’, and therefore, many of these harms are avoidable.

Accidents were deemed worthy of reporting when caused by an external presence, such as an animal, rather than by staff themselves. This suggests that blame attribution may be perceived as a requirement for reporting, which supports the findings in other industries that events construed as ‘personal failures’ were less worthy of reporting than events with an external cause [12]. Injury self-attribution may be deemed embarrassing, unworthy of sharing and unlikely to lead to future behaviour change for others. A culture of injury ‘ownership’ needs to be embraced, which could facilitate more rapid change in risk mitigation and reporting strategies.

Needlestick injuries were a common incident described as not worthy of reporting, likely because they did not fulfil the ‘injury’ criteria: they were perceived as common, minor, self-attributed and did not cause lasting pain. However, needlestick injuries are harmful, and it was concerning to see that they were considered to be an everyday occurrence [17–20]. This is consistent with previous research which described veterinary attitudes to needlestick injuries as being ‘relatively lax’ [5].

The results indicate that the veterinary industry should take note of the discrepancy of perception of what constitutes work-related injury, in its practices and reporting protocols. Managers need to recognize that there is a clear differentiation in injury perception between veterinary sectors and job roles. Yet acknowledging these differences to their staff could widen that divide and imply that certain harms are inevitable in some situations. We suggest that it is important that each workplace considers: the culture and attitudes of its employees; what is thought to be an ‘everyday’ occurrence; what is perceived as ‘acceptable risk’ and how the organization plans to manage its reporting standards. Managers need to consider what their local workforce considers an acceptable level of risk; what is considered a workplace injury; what is deemed unworthy of reporting; whether staff feel comfortable or have time to report safety breaches and whether staff feel there are useful consequences from reporting. Close attention to the human factors within the microculture of each workplace is likely to bring about meaningful change and improve worker safety.

Numerous veterinary professionals will only report a severe injury, and therefore, reporting statistics will underestimate the true prevalence. Equine and production animal veterinarians have a high threshold before acknowledging an incident is a work-related injury. The discordance in definition needs to be accounted for when interpreting injury reporting figures. Industry-wide cultural change needs to occur to support individuals in appreciating the need for reporting and collating injury data. This has occurred in other industries; a hospital found that an anonymous reporting system increased the reporting of incidents by over five times [21]. This aligns with other research which supported ‘identification and blame’ as a significant barrier to reporting [22]. The need for reporting systems to be presented in ways which match the culture and concerns of the workers, concerning blame attribution and promoting inter-staff harmony is highlighted.

There are clear limitations to this study. The number of respondents in equine and production animal practice was low. The reasons for this are unknown, but could be due to a range of factors. The profession receives frequent surveys from employers, governing bodies and researchers; subsequently, there could be survey fatigue. There may have been a lack of opportunity as equine and production animal veterinarians primarily work in settings that do not facilitate responding to surveys (i.e. farms, stables, in a vehicle between jobs), especially surveys with the potential for descriptive answers. The survey topic may have dissuaded this demographic from taking part. As seen in the results, they have a high threshold for what constitutes an injury and may see workplace injuries as a minor issue, and therefore may not want to participate in this research topic. Second, since the survey was disseminated via the research funder, participants might have provided biased answers writing what they felt that their employers ‘expected’. This is potentially highlighted by the high number of respondents answering that all injuries should be reported. However, the influence was hopefully minimized by reiterating that responses would not be seen or analysed by the funder and that all responses were anonymous. Finally, the survey methodology led to predominantly short-answer descriptions; while these provide valuable insight, additional information about context and experience would be invaluable in understanding attitudes to reporting and the broader cultural contexts. Though inductive content analysis is appropriate in a survey with short responses, an absence of mentioning a code does not mean that it lacks importance for those individuals. To explore the differences in attitude versus practice meaningfully, more in-depth observational or ethnographic study is required.

This study highlights that defining a veterinary work-related injury was a complex and nuanced concept. Each veterinary sector has its own culture of risk and injury expectations. These expectations can impact what people consider ‘everyday’ risks, which are not worth reporting. Veterinary practices should explore their microcultures to accidents, injuries and reporting practices. Care must be taken interpreting injury statistics as it is likely that under-reporting levels are high, with only the most serious injuries being reported. These data suggest that the most serious injuries may be reported well and could be representative of the most traumatic incidents within the veterinary industry. We suggest that an individualised approach will lead to targeting appropriate endpoints: some workplaces may need to work on revisiting their ideas around acceptable risk, whilst others may be good at minimizing work-related harm, but have a complex or blame-associated reporting system. Fostering a work culture, that supports ‘ownership’ of an injury, at a local level is most likely to be successful in creating meaningful change in attitude and behaviour.
